# How Leisure Involvement Affects Repurchase Intention in Fitness Clubs? The Mediating Role of Commercial Friendship

**DOI:** 10.3389/fspor.2022.777185

**Published:** 2022-04-29

**Authors:** Yanting Wang, Yi Gao, Fong-Jia Wang

**Affiliations:** ^1^Department of Physical Education, College of Sports and Recreation, National Taiwan Normal University, Taipei, Taiwan; ^2^School of Physical Education, Quanzhou Normal University, Taipei, Taiwan; ^3^Institute of Tourism and Leisure Mangement, Fujian Business University, Taipei, Taiwan; ^4^Tamkang University, New Taipei, Taiwan

**Keywords:** customized fitness consumption, leisure involvement, repurchase intentions, commercial friendship, mediated effect, fitness services

## Abstract

This study explores the relationships among leisure involvement, business or commercial friendship, satisfaction, and willingness to repurchase in customers' use of customized fitness services. This study randomly sampled and analyzed the customers of fitness clubs (*N* = 234) in China. The results showed that leisure involvement had a positive impact on repurchase intention and satisfaction. Moreover, the mediating effect of commercial friendship was found in the relationship between leisure involvement, repurchase intention, and satisfaction. Overall, the study contributes to the literature by exploring the interaction effects of different types of consumer leisure involvement and commercial friendships in customized fitness services models on satisfaction and repurchase intentions'.

## Introduction

There has been an increase in people's interest in fitness and the number of fitness enterprises associated with physical activity, which could benefit society. Not only have people started paying attention to physical activities and fitness, but their fitness demands are also diversifying (Angosto et al., 2020). Due to these trends, the fitness industry has become the representative product of consumption upgrading (Baker et al., 2018).

The new fitness club model is becoming highly prominent. It has the main characteristics of human and technology interaction; data-intensive, customized optimization; and participation by a wider network community (Parasuraman et al., 1985; Baena-Arroyo et al., 2020; Ivens et al., 2020). Appearing in new market modes (i.e., private gym training studios and internet smart gyms), customized fitness services have become the main point of operating profit growth in the fitness industry due to their high prices (Angosto et al., 2020). Specifically, customized fitness services are offerings where private fitness coaches customize fitness plans and chart healthy diet recipes that meet customers' needs and provide frequent feedback and personalized guidance (Lovelock, 1983). Customized services are part of merchants' promotion strategies, which are regarded as the main strategies affecting customer behavior (Montgomery and Smith, 2009). The positive emotions of customers after enjoying the customized services affect the customers' repurchase intentions (Pappas et al., 2014). In fitness centers, customers must be physically present and exercise actively for the service delivery process to be considered successful (Chiu et al., 2015). Thus, during the process of training and guidance, private fitness coaches get into direct contact with customers to encourage the latter's participation, which requires the establishment of a close and trusting commercial friendship between the coaches and the customers.

Leisure involvement represents how an individual and the external stimulus are related (Baker et al., 2018). Therefore, leisure involvement can be considered as the extent to which an individual is involved in leisure and recreational activities. Moreover, the degree of customers' leisure involvement inevitably affects their fitness outcomes, such as increasing physical health or losing weight (Ramaswamy, 2009). Meanwhile, the process of forming a commercial friendship could create adverse feelings that harm the commercial friendship, leading to customer dissatisfaction (Baker et al., 2018). However, whether it also affects customer satisfaction has not yet been supported by empirical research. Therefore, the effects of commercial friendships on customer behaviors, such as leisure involvement and customer satisfaction, need to be explored.

Given the advantages of customer relationship management and customers' leisure involvement for consumers in the fitness industry and using this as the theoretical basis, this study seeks to explore the relationships among leisure involvement, business or commercial friendship, satisfaction, and willingness to repurchase in customers' use of customized fitness services. This study contributes to the literature in various ways by exploring the interaction effects of different types of consumer leisure involvement and commercial friendships in customized fitness services models on satisfaction and repurchase intentions'.

## Hypotheses Development

### Relationship Between Leisure Involvement, Satisfaction and Repurchase Intentions

Leisure involvement is a psychological state generated during the process of interaction between the individuals and the recreation activities, recreation destinations, and related recreation products; it is a behavior-driven feature (Parasuraman et al., 1988; Havitz and Dimanche, 1997; Rosenbaum, 2009). Leisure involvement is a multifaceted conceptualization, including three-facet solution comprised of attraction, centrality, and self-expression (McIntyre, 1989; McQuarrie and Phillips, 2005). Attraction reflects hedonic value and the enjoyment derived from an activity. Centrality relates to how central the activity is to the individual's daily life. Self-expression refers to the self-representation or the impression of the self that individuals wish to convey to others through their leisure participation (Kyle and Chick, 2004).

During the 1980s, Sherif and Hovland's work was extended to consumer behavior research to understand the purchasing behavior of consumer goods (Laurent and Kapferer, 1985) and has since been applied to leisure contexts. Scholars have found that leisure involvement positively influences the effects of consultations and communications via social networks and interpersonal relationships (Lin et al., 2017) and is positively related to place attachment and the identity with of the destinations (Shen et al., 2008). In one study, the leisure involvement of bicycle users was found to be positively related to place attachment (Li et al., 2012). Johnson et al. (2006) found that couple leisure involvement, leisure satisfaction, and marital relationship satisfaction were positively correlated in their study. Further, Sato et al. (2014) reported that a single construct of leisure involvement was positively associated with life domain satisfaction.

In view of the literature, satisfaction has been widely used in various academic fields to measure consumers' views, cognition and behavioral performance of products and services, work, living environment and outdoor recreation quality, and has also become a common indicator to measure consumer behavior. Westbrook and Oliver (1981) argued that satisfaction is an emotion, which is a kind of psychological state in which the expectations are consistent with the actual, experienced feelings. In the final step of the satisfaction formation process, satisfaction determines the intentions of whether to patronize a store in the future. Customer satisfaction is the antecedent of customers “repurchasing of services (Fornell, 1992; Szymanski and Henard, 2001)”.

Despite the wealth of literature, the concept of leisure involvement has rarely been studied in the field of fitness (Havitz et al., 2013). Under the premise that fitness has become a lifestyle, we expect that the three facets of leisure involvement in the activity will differently address psychological needs that promote satisfaction with fitness center services.

Given that each involvement facet has a distinctive meaning, For instance, fitness attraction could contribute to a detachment from work; fitness as centrality might be more associated with an affiliation with family or friends; and self-expression could be equated to the pursuit of the meaning in life. To examine whether the leisure involvement of the customers of customized fitness services is related to their satisfaction and repurchase intentions, our study puts forward the following hypotheses:

***Hypothesis 1 (H1):*** Leisure involvement positively affects satisfaction.
***Hypothesis 2 (H2):*** Leisure involvement positively affects repurchase intentions.

### Commercial Friendshipas a Mediator

Commercial friendships refer to a gradual development of friendship between the service providers and the customers in the service industry through cooperation. Commercial friendships are characterized by expressiveness, instrumentality, and cooperation, as well as the sharing of information (Goodwin and Gremler, 1996; Wang et al., 2017). Commercial friendships between the service providers and the customers are mutually beneficial (Wu, 2000; Chen et al., 2005; Tsai and Huang, 2007). Commercial friendship has been found to have a positive impact on service satisfaction, loyalty, and public extolment (Kelley and Hoffman, 1997). It is also associated with promoted service personalization and transactions and improved satisfaction and public praising (Price and Arnould, 1999).

**Figure 1:**
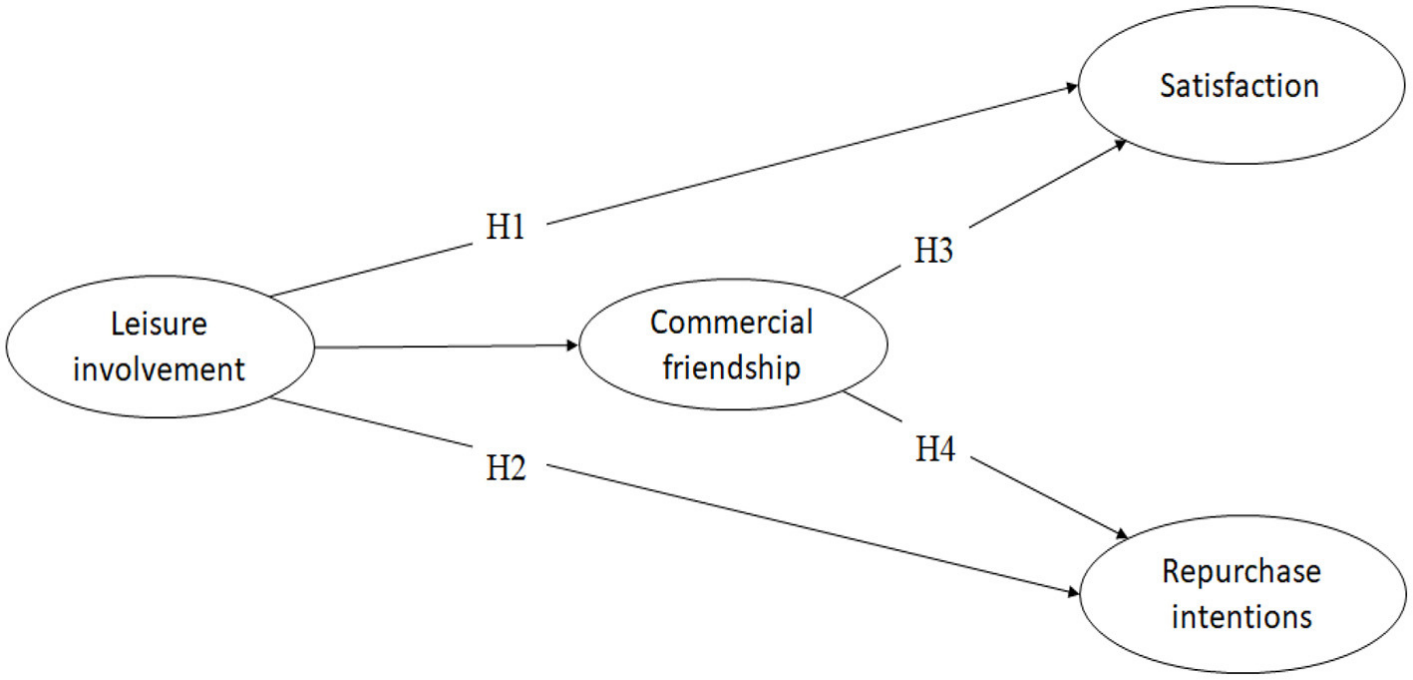
Hypotheses model.

Numerous studies have suggested that the level of leisure involvement is positively linked to activity and equipment knowledge, frequency of participation, intensity of participation, and duration of participation (Havitz et al., 2013; Rosenbaum et al., 2015). Leisure involvement can be considered the extent to which an individual is involved in leisure and recreational activities. Thus, service providers establish trust with customers, and customers focus on feelings and become perceptual purchase decision-makers (Johnson et al., 2011). In the fitness service industry, the production and consumption of services during the interactions between private fitness coaches and customers proceed simultaneously and cannot be separated. For example, commercial friendships between service providers and customers affect customer service satisfaction and the service quality (Price and Arnould, 1999). Similarly, the establishment of commercial friendships between the customers and the employees of fitness enterprises not only exerts a positive impact on customer loyalty but also causes the customers to identify with the enterprises, resulting in word-of-mouth promotion and repurchase intentions (Price and Arnould, 1999). In other words, commercial friendship may impact the relationship between leisure involvement and service satisfaction and the relationship between leisure involvement and repurchase intentions. Therefore, we put forward the following hypotheses (Figure 1):

***Hypothesis 3 (H3):*** Commercial friendship mediates the relationship between leisure involvement and satisfaction.
***Hypothesis 4 (H4):*** Commercial friendship mediates the relationship between leisure involvement and epurchase intentions.

## Methods

### Participants and Procedures

Participants were recruited from three city's fitness clubs across China, in particular Quanzhou, such as Shuhua fitness management company, Center of Power Castle Fitness Management, and Zhong Kong City Fitness Center, which were selected to represent different marketing areas. It should be noted that the permission to conduct the survey was approved by all fitness centers. Prior to data collection, ethical approval was obtained from the first author's university. Moreover, all participants were asked to sign and submit consent forms, and their responses would remain confidential and used for research purposes only. This study received 250 questionnaires but 16 surveys were discarded because of incomplete responses. As a result, there were a total 234 useable surveys, which were distributed to final sample. Of the total participants, most of them are male (56.6%, *n* = 456) with an average age of 37.2 years (SD = 13.8). Moreover, the majority of respondents education degree college (43.1%, *n* = 101) (Table 1).

**Table 1:** Demographic information of the participants.

**Characteristics**	***N***	**%**	**Characteristics**	***N***	**%**
**Gender**			**Age**		
Male	89	38	<20	7	3.0
Female	145	62	21–30	144	61.6
			31–40	78	33.3
			More than 41	5	2.1
**Income**			**Education**		
<3,000	20	8.5	Junior high or below	12	5.1
3,001–5,000	43	18.4	Senior high school	30	12.9
5,001–7,000	51	21.8	Vocational school	81	34.6
7,001–9,000	28	12	College/university	101	43.1
More than 9,001	92	39	Graduate and above	10	4.3

*N = 229*.

### Measurement

#### Leisure Involvement Scale

The scales developed by scholars, such as Kyle and Mowen (2005) and McQuarrie and Phillips (2005), were mainly used for developing the leisure involvement scale and facilitating the understanding of the investigators. The scale was revised to suit the fitness field. The scale included three dimensions, namely attraction (four questions), centrality (five questions), and self-expression (three questions). Items were rated on 7-point Likert scales ranging from 1 (strongly agree) to 7 (strongly disagree). The internal consistency and the item-total correlation analysis methods were used. The Pearson product-moment coefficient was between 0.53 and 0.91, and the critical ratio (CR), value reached a significant level. Exploratory factor analysis was implemented, and the principal component analysis and varimax rotation method were used for testing. The Kaiser-Meyer-Olkin (KMO) value of the leisure involvement scale was 0.932, and the Barlett sphericity test value was 1733.33, with *p* < 0.000. By selecting factors with a factor load of 0.5 or more, the total explained variance reached 84.036%. Cronbach's α was used to verify the internal consistency of the scale; Cronbach's α values for the three factors of attraction, centrality, and self-expression were 0.95, 0.94, and 0.89, respectively, and Cronbach's α of the leisure involvement scale was 0.96, indicating good reliability and validity.

#### Commercial Friendship Scale

Referencing the scales developed by McQuarrie and Munson (1987), Price and Arnould (1999), and other scholars for the hairdressing and club service industries, this study redesigned the commercial friendship scale to include dimensions like friendship (four questions), trust (three questions), and establishment of personal relationships (three questions). A seven-point Likert-type scale was used as a scoring method, with the highest score of seven indicating high consistency with the expression and the lowest score of one indicating high inconsistency with the expression. Exploratory factor analysis showed that the KMO value of the commercial friendship scale was 0.852, and the Barlett sphericity test value was 1697.661, with *P* < 0.000. The factors with a factor load of 0.5 or more were selected. As the question items of friendship and trust were similar, they were ultimately combined into the dimension of friendliness, excluding one question as a result. Two factors, namely, friendliness and the establishment of personal relationships, were extracted, and the total explained variance reached 89.78%. Cronbach's α values for friendliness and the establishment of personal relationships were 0.89 and 0.96, respectively, and Cronbach's α of the commercial friendship scale was 0.97, showing good reliability and validity.

#### Satisfaction Scales

Referring to the satisfaction scales of Chen et al. (2005), Oliver (1980), Saha Gour and Theingi (2009), and other scholars for the tourism and catering industries, this study revised the satisfaction scale to include six questions. A seven-point Likert-type scale was used as a scoring method, with the highest score of seven indicating high consistency with the expression and the lowest score of one indicating high inconsistency with the expression. The results showed that the KMO value of the satisfaction scale was 0.704, and the Barlett sphericity test value was 472.961, with *P* < 0.000. The total explained variance was 90.16%, and Cronbach's α of the satisfaction scale was 0.96, showing good reliability and validity.

#### Repurchase Intention Scales

Referring to the repurchase intention scales of Hu and Wu (2009), and other scholars for the home delivery and tourism industries, this study revised the expressions of the fitness services in the repurchase intention scale to include three items: “I am likely to buy the fitness course service of the current personal trainer again,” “I will purchase customized fitness services through online booking under economic permission,” and “I will purchase customized fitness services through online booking under time permission.” A seven-point Likert-type scale was used as a scoring method, with the highest score of seven indicating high consistency with the expression and the lowest score of one indicating high inconsistency with the expression. The results showed that the KMO value of the repurchase intention scale was 0.704, and the Barlett sphericity test value was 472.961, with *P* < 0.000. The total explained variance was 90.16%, and the Cronbach's α of the repurchase intention scale was 0.94, showing good reliability and validity.

### Data Processing

The data analysis included the following critical stages. The data were analyzed via descriptive statistics, correlations, factor analysis, multivariate hierarchical regression analysis, and Sobel test (Hayes, 2018) using the Statistical Package for the Social Sciences (SPSS version 20.0; SPSS Inc., Chicago, IL).

## Results

### Correlation Analysis Among the Main Variables

The Pearson relative analysis method was used, and the correlation matrix between the study variables is shown in Table 2. There was a significant correlation among leisure involvement, commercial friendship, satisfaction, and repurchase intentions. The study of the correlation among the variables provided a good research basis for the subsequent test of intermediary effects.

**Table 2:** Summary of the descriptive statistics and correlation analysis results among the variables.

	**M ±SD**	**Leisure involvement**	**Commercial friendship**	**Repurchase intentions**	**Satisfaction**
Leisure involvement	2.65 ± 1.19				
Commercial friendship	2.69 ± 1.07	0.79^**^			
Repurchase intentions	2.38 ± 1.22	0.79^**^	0.78^**^		
Satisfaction	2.23 ± 1.156	0.79^**^	0.81^**^	0.83^**^	–

* *p <0.05*;

** *p <0.01*.

### Model Validation Factor Analysis

Multivariate hierarchical regression analysis was adopted, and before the regression analysis, a multicollinearity diagnosis of the data was conducted. Hellier Phillip et al. (2003) have pointed out that the variance inflation factor (VIF) value must be lower than 10 to avoid serious multicollinearity problems (Hair et al., 1995). In this study, the VIF values of all variables were less than four, implying that the multicollinearity problem did not exist. The model summary and the parameter estimation results obtained by the multivariate hierarchical regression method are shown in Table 2. All three variables of leisure involvement, satisfaction, and commercial friendship had significant implications for repurchase intentions. It can be seen from the change in the determination coefficient that satisfaction alone (Model 1) could explain 69.4% of the variation of the repurchase intentions, which supports H3 of this study. In Model 2, the newly added leisure involvement variable could significantly increase the explanatory variation by 4.1%. In Model 3, commercial friendship could further increase the explanatory variation by 1.1%. These three predictive variables could explain 74.6% of the variance of satisfaction. The standardized regression coefficient of satisfaction was the largest (β = 0.46), indicating that it had greater explanatory power. It can be seen from the positive and negative values of the standardized regression coefficient that leisure involvement, commercial friendship, satisfaction, and repurchase intentions were positively correlated.

### Model Intermediary Effect Test

The intermediary effect was calculated by multiplying the Sobel test coefficients (product of coefficients, a^*^b). Sobel's Z-value test and the 95% confidence interval test of the bootstrapping method were used for the intermediary effect test to cross-validate whether the intermediary effect was significant. As shown in Table 3, leisure involvement had a significant direct effect on satisfaction (standardized effect value = 0.77, *p* < 0.01), thereby supporting H1. In addition, leisure involvement had a significant direct effect on repurchase intentions (standardized effect value = 0.81, *p* < 0.01), supporting H2.

**Table 3:** Summary of the results of the multivariate hierarchical regression of each variable explaining repurchase intentions.

	**Model 1**		**Model 2**		**Model 3**	
	**Non-standardized coefficient B (standardization coefficient)**	***t***	**Non-standardized coefficient B (standardization coefficient)**	***t***	**Non-standardized coefficient B (standardization coefficient)**	***t***
(Constant)	0.41	3.21^**^	0.13	0.94	−0.04	−0.29
Satisfaction	0.88 (0.83)	17.38^**^	0.59 (0.56)	7.56^**^	0.49 (0.46)	5.57^**^
Leisure involvement			0.35 (0.35)	4.67^**^	0.27 (0.26)	3.24^**^
Commercial friendship					0.24 (0.21)	2.55^**^
*R* ^2^	0.696		0.739		0.752	
Adjusted *R*^2^	0.694		0.735		0.746	
*R*^2^ change	0.696		0.043		0.012	
*F*-value	302.136		21.845		6.523	

^*^p <0.05;

** *p <0.01*.

**Figure 2:**
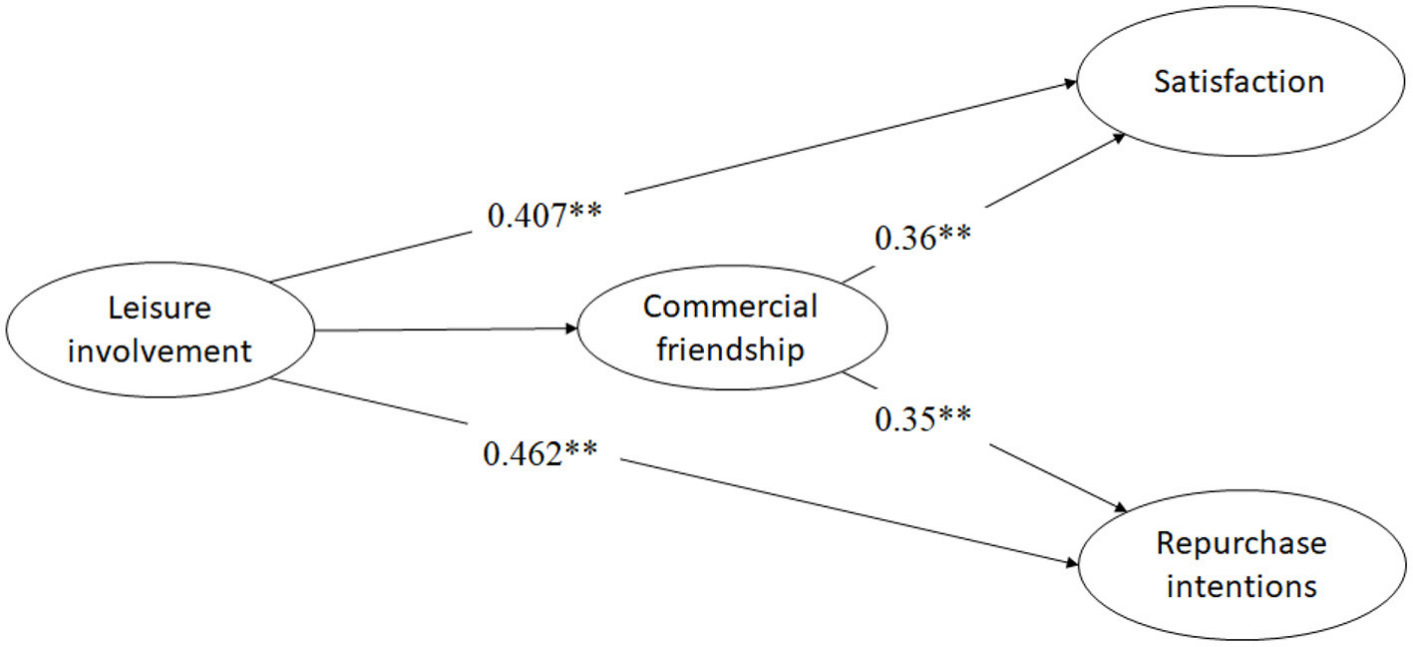
The intermediary effect model of commercial friendship among leisure involvement, satisfaction, and repurchase intentions. **p < 0.01.

Furthermore, under the control of leisure involvement, commercial friendship had a significant effect on satisfaction (standardized effect value = 0.51, *p* < 0.01), which supports H3. The indirect effect of leisure involvement on satisfaction through commercial friendship was 0.36, and the normal test value of the indirect effect was *z* 1 = 5.725, *p* < 0.01, showing a significant indirect effect (Table 4). In other words, commercial friendship played a partial intermediary role between leisure involvement and satisfaction, which supports H3. The indirect effect of leisure involvement on repurchase intentions through commercial friendship was 0.35, and the normal test value of the indirect effect was *z* 2 = 5.026, *p* < 0.01, showing a significant indirect effect. In other words, commercial friendship played a partial intermediary role between leisure involvement and repurchase intentions, which supports H4 (Figure 2).

**Table 4:** Test results of the intermediary effects of commercial friendship.

**Effect**	**Impact path**	**Standardized effect value**	**Standard deviation**	***t***
Direct effect	X → Y1	0.771	0.051	15.174^**^
	X → M	0.716	0.048	15.046^**^
	M.X → Y1	0.508	0.082	6.204^**^
	X.M → Y1	0.407	0.074	5.504^**^
Indirect effect		0.364	0.064	4.32^**^
Direct effect	X → Y2	0.809	0.055	14.851^**^
	X → M	0.716	0.048	15.046^**^
	M.X → Y2	0.484	0.091	5.344^**^
	X.M → Y2	0.462	0.082	5.662^**^
Indirect effect		0.347	0.069	3.45^**^

^*^p <0.05;

** *p <0.01*.

*(X) leisure involvement, (M) commercial friendship, (Y1) satisfaction, (Y2) repurchase intentions*.

## Discussion

### Relationships Among the Leisure Involvement, Satisfaction, and Repurchase Intentions of Customized Fitness Customers

This study explores the relationships among the leisure involvement, satisfaction, and repurchase intentions of the customers of customized fitness services, as well as the role of commercial friendship in leisure involvement's relationships with satisfaction and repurchase intentions.

The results showed that leisure involvement had a significant direct effect on satisfaction and asignificant direct effect on repurchase intentions. Further, commercial friendship played a partial intermediary role between leisure involvement and satisfaction. Hillsdon (2001) confirmed that higher frequency leisure participation is associated with a lower likelihood of abandonment (Oliver, 1980). Therefore, fitness enterprises should strengthen the leisure involvement of fitness customers. For example, the managers of fitness centers could offer a variety of services and/or services with durations of more than 1 h (Hillsdon, 2001). Fitness enterprises could improve the visiting rate and participation of fitness customers by establishing member exercise reward systems that would make the fitness enterprise more attractive to the customers (Clavel San Emeterio et al., 2016).

Further, private fitness coaches can improve customers' leisure involvement by providing customized fitness services, which is more conducive than general fitness services salesto improving customer satisfaction and increasing the repurchase intentions of customers. In the process of providing customized fitness services, private fitness coaches should focus on the self-expression of the fitness customers while formulating personalized exercise plans, determining the training intensity and density, and taking into account both diet and nutrition. Moreover, in providing guidance and interactive exercise plans, the fitness coaches should guide the customers through language and behavior to increase the latter's attention to fitness, establishing the central position of fitness in their lives. Through the diversification of exercise plans, the attraction of the customized fitness customers to fitness can be enhanced, thereby raising the customers' leisure involvement as well.

### Intermediary Effect of Commercial Friendship

This study revealed that leisure involvement can affect the satisfaction and repurchase intentions of customized fitness consumers directly and indirectly through the intermediary effect of commercial friendship. Past research confirms have showed that the commercial friendship between customized fitness consumers and private fitness coaches is positively correlated with and could predict satisfaction and repurchase intentions (Price and Arnould, 1999; Partridge et al., 2014). The interaction and communication among customers and between customers and enterprises can be increased by conducting activities, such as parties and outdoor activity days. The primary goal is to foster an emotional and psychological bond between customers and the firm (Wang et al., 2017). Enterprises can establish a fitness customer column in their virtual social media networks, through which customers can express their fitness experience and needs, thereby building an online community of commercial friendship. In this way, the community of commercial friendship cultivated by fitness enterprises would allow customers to develop friendships, encourage each other, exercise on time, and increase the degree of leisure involvement.

The commercial friendship between customers and private fitness coaches is a result of the economic and social exchanges between them and forms an important part of the relationship marketing of fitness enterprises (Rosenbaum et al., 2015). The commercial friendship between customers and private fitness coaches is also a relationship asset of fitness enterprises that is difficult for competitors to imitate and replace. At times, customized fitness consumers may no longer have the intention to repurchase due to such factors as a change of residence, change of jobs, and access to other leisure modes (Wang and Guo, 2006; Withall et al., 2011).

The strength of relatedness as commercial friendship captures both the social and service benefits of direct interactions between actors. Commercial friendship is a business-based relationship, built on affection, intimacy, and acts of social support developed over time (Withall et al., 2011). Given that results, the role of commercial friendship is to enhance the interactions and service production between fitness customers and service providers (i.e., fitness coaches), specifically when building long-term relationships (Garzaniti et al., 2011). In fitness context commercial friendship relationships, customers pursue not only direct service outcomes, but also look for interpersonal interactions with service providers to realize relational benefits that lead to greater satisfaction and repurchase intention (Wang et al., 2017).

In addition, noted that once people are active, high levels of social interaction, interest, and enjoyment are all associated with improved levels of retention. Thus, strengthening the interpersonal skills of private fitness coaches and the development of good friendships between the fitness customers and the coaches can increase customer retention. Role-playing can be used to simulate the interactions between fitness customers and private fitness coaches, in which the private coaches play the role of the fitness customers, deal with problems from the standpoint of the customers, and experience the feelings and needs of the customers. Therefore, fitness enterprises can change the service concept from customer first to customer as a friend and create a more humane fitness consumption environment for customers (Shain and Chalasani, 1992). In the process of interacting with customers, private fitness coaches should listen to the customers, pay attention to the inner feelings of the customers, adjust exercise programs and communication methods in time, increase the frequency of effective communication, and promote commercial friendships.

### Limitations and Future Directions

This study has certain limitations. First, in this study, as the customized fitness consumers of specific fitness clubs were included as the study group, the study results do not encompass all customized consumption groups of fitness and leisure services. Thus, follow-up studies are suggested to include different types of customized fitness and leisure service groups, such as competition consumers and outdoor sports consumers, to reassess the applicability of the research framework. Second, this study was a cross-sectional study, which was limited in its ability to track the subtle changes in the relationship between fitness consumers and private fitness coaches continuously. This study can't make causal inferences, and future directions will increase experimental control. Finally, all variables were collected at the same time point, which could have led to common method variance (CMV), because of which causal inferences could not be derived. Therefore, it is suggested that follow-up studies adopt the time-interval method to collect subscale data to reduce the causal confusion caused by CMV.

## Conclusion

In this study, the results showing the positive effects of consumer leisure involvement and commercial friendships on customers' use of customized fitness services were revealed to have good reliability and validity, suggesting that they could be used for the promotion of customized fitness services. In addition to clarifying the relationships among leisure involvement, satisfaction, and repurchase intentions, this study discussed the intermediary mechanism of commercial friendship that could predict the effects of leisure involvement on the satisfaction and repurchase intentions of customized fitness customers. Overall, the results of this study contribute to the literature in various ways by exploring the interaction effects of different types of consumer leisure involvement and commercial friendships in customized fitness services models on leisure involvement outcomes. At the same time, these findings have allowed the research team to identify a range of recommendations for sports organizations and researchers, which will help them to address future studies and thus cultivate the growth of the evaluation of commercial friendship in the fitness industry.

## Data Availability Statement

The raw data supporting the conclusions of this article will be made available by the authors, without undue reservation.

## Author Contributions

YW and F-JW: conceptualization. F-JW: methodology, writing-original draft preparation, and writing—review and editing. YW and YG: formal analysis and investigation. YW: data curation, funding acquisition, and project administration. All authors have read and agreed to the published version of the manuscript.

## Conflict of Interest

The authors declare that the research was conducted in the absence of any commercial or financial relationships that could be construed as a potential conflict of interest.

## Publisher's Note

All claims expressed in this article are solely those of the authors and do not necessarily represent those of their affiliated organizations, or those of the publisher, the editors and the reviewers. Any product that may be evaluated in this article, or claim that may be made by its manufacturer, is not guaranteed or endorsed by the publisher.

## References

[B1] Angosto S. García-Fernández J. Valantine I. Grimaldi-Puyana M. (2020). The intention to use fitness and physical activity apps: a systematic review. Sustainability 12, 6641. 10.3390/su12166641

[B2] Baena-Arroyo M. J. García-Fernández J. Gálvez-Ruiz P. Grimaldi-Puyana M. (2020). Analyzing consumer loyalty through service experience and service convenience: differences between instructor fitness classes and virtual fitness classes. Sustainability 12, 828. 10.3390/su12030828

[B3] Baker B. J. Jordan J. S. Funk D. C. (2018). Run again another day: the role of consumer characteristics and satisfaction in repeat consumption of a sport-related experience product. J. Sport. Manage. 32, 38–52. 10.1123/jsm.2017-0042

[B4] Chen Y. Li H. Lin A. (2005). Influence of social friendship on customers' willingness to reuse in customized service: a case study of clubhouse Industry. Market. Rev. 2, 463–489.

[B5] Chiu W. Kwag M.-S. Bae J.-S. (2015). Customers as partial employees: the influences of satisfaction and commitment on customer citizenship behavior in fitness centers. J. Phys. Educ. Sport 15, 627. 10.7752/jpes.2015.04095

[B6] Clavel San Emeterio I. Iglesias-Soler E. Gallardo L. Rodriguez-Cañamero S. García-Unanue J. (2016). A prediction model of retention in a Spanish fitness centre. Manag. Sport Leisure 21, 300–318. 10.1080/23750472.2016.1274675

[B7] Fornell C.. (1992). A national customer satisfaction barometer: the Swedish experience. J. Mark. 56, 6–21. 10.1177/002224299205600103

[B8] Garzaniti I. Pearce G. Stanton J. (2011). Building friendships and relationships: The role of conversation in hairdressing service encounters. Manag. Serv. Qual. 21, 667–687. 10.1108/09604521111185646

[B9] Goodwin C. Gremler D. D. (1996). Friendship over the counter: how social aspects of service encounters influence consumer service loyalty. Adv. Serv. Market. Manage. 5, 247–282. 10.1016/S1067-5671(96)05059-7

[B10] Hair J. F. Anderson R. E. Tatham R. L. Black W. C. (1995). Multivariate Data Analysis With Readings, 4th Edn.. Englewood Cliffs, NJ: Prentice Hall.

[B11] Havitz M. E. Dimanche F. (1997). Leisure involvement revisited: conceptual conundrums and measurement advances. J. Leisure Res. 29, 245–278. 10.1080/00222216.1997.11949796

[B12] Havitz M. E. Kaczynski A. T. Mannell R. C. (2013). Exploring relationships between physical activity, leisure involvement, self-efficacy, and motivation via participant segmentation. Leisure Sci. 35, 45–62. 10.1080/01490400.2013.739890

[B13] Hayes A. F.. (2018). Partial, conditional, and moderated mediation: Quantification, inference, and interpretation. Commun. Monogr. 85, 4–40. 10.1080/03637751.2017.1352100

[B14] Hellier Phillip K. Geursen Gus M. Carr Rodney A. Rickard John A. (2003). Customer repurchase intention: a general structural equation model. Eur. J. Market. 37, 1762–1800. 10.1108/03090560310495456

[B15] Hillsdon M.. (2001). Winning the Retention Battle. London: Fitness Industry Association.

[B16] Hu K. J. Wu X. Y. (2009). The impact of brand image and brand alliance fit on consumer purchase intention of home furnishing industry. J. Chinese Manage. Rev. 12.

[B17] Ivens B. Riedmueller F. van Dyck P. (2020). Success factors in managing the sponsor–sponsee relationship—a fuzzy-set qualitative comparative analysis for state-owned enterprises in Germany. Int. J. Sports Market. Sponsorship 21, 577–596. 10.1108/IJSMS-09-2019-0102

[B18] Johnson B. R. Ross W. T. Coulter R. (2011). Expressive oriented relationships: a new type of commercial friendships. Adv. Consum. Res. 39, 757–758.

[B19] Johnson H. A. Zabriskie R. B. Hill B. (2006). The contribution of couple leisure involvement, leisure time, and leisure satisfaction to marital satisfaction. Marriage Fam. Rev. 40, 69–91. 10.1300/J002v40n01_05

[B20] Kelley S. W. Hoffman K. D. (1997). An investigation of positive affect, prosocial behaviors and service quality. J. Retail. 73, 407–427. 10.1016/S0022-4359(97)90025-7

[B21] Kyle G. Chick G. (2004). Enduring leisure involvement: the importance of personal relationships. Leisure Stud. 23, 243–266. 10.1080/0261436042000251996

[B22] Kyle G. T. Mowen A. J. (2005). An examination of the leisure involvement—agency commitment relationship. J. Leisure Res. 37, 342–363. 10.1080/00222216.2005.11950057

[B23] Laurent G. Kapferer J.-N. (1985). Measuring consumer involvement profiles. J. Market. Res. 22, 41–53. 10.1177/002224378502200104

[B24] Li R. Wen Y.- H. Huang L.- B. (2012). Study on leisure involvement and place attachment of bicycle path users – a case study of Puzixi bicycle Path. J. Leisure Sports 11, 1–13.

[B25] Lin Y.-Q. Ni M.-L. Chen Y.-T. (2017). Empirical study on the influence of information transmission effect, interpersonal relationship and leisure involvement on the recovery of forest tourism perception: a case study of Luan Mountain Forest Culture Museum in Taitung County. J. Tourism Leisure Manage. 5, 1–13.

[B26] Lovelock C. H.. (1983). Classifying services to gain strategic marketing insights. J. Mark. 47, 9–20. 10.1177/002224298304700303

[B27] McIntyre N. . (1989). The personal meaning of participation: enduring involvement. J. Leisure Res. 21, 167–179. 10.1080/00222216.1989.11969797

[B28] McQuarrie E. F. Munson J. M. (1987). “The zaichkowsky personal involvement inventory: Modification and extension,” in Advances in Consumer Research, Vol. 14, eds M. Wallendorf and P. Anderson (Provo, UT: Association for Consumer Research), 36–40.

[B29] McQuarrie E. F. Phillips B. J. (2005). Indirect persuasion in advertising: How consumers process metaphors presented in pictures and words. J. Advert. 34, 7–20. 10.1080/00913367.2005.10639188

[B30] Montgomery A. L. Smith M. D. (2009). Prospects for personalization on the internet. J. Interact. Market. 23, 130–137. 10.1016/j.intmar.2009.02.001

[B31] Oliver R. L.. (1980). A cognitive model of the antecedents and consequences of satisfaction decisions. J. Market. Res. 17, 460–469. 10.1177/002224378001700405

[B32] Pappas I. O. Pateli A. G. Giannakos M. N. Chrissikopoulos V. (2014). Moderating effects of online shopping experience on customer satisfaction and repurchase intentions. Int. J. Retail Distrib. Manage. 42, 187–204. 10.1108/IJRDM-03-2012-0034

[B33] Parasuraman A. Zeithaml V. A. Berry L. L. (1985). A conceptual model of service quality and its implications for future research. J. Mark. 49, 41–50. 10.1177/002224298504900403

[B34] Parasuraman A. Zeithaml V. A. Berry L. L. (1988). Servqual: A multiple-item scale for measuring consumer perc. J. Retail. 64, 12.

[B35] Partridge J. A. Knapp B. A. Massengale B. D. (2014). An investigation of motivational variables in CrossFit facilities. J. Strength Condit. Res. 28, 1714–1721. 10.1519/JSC.0000000000000288 24149755

[B36] Price L. L. Arnould E. J. (1999). Commercial friendships: service provider–client relationships in context. J. Mark. 63, 38–56. 10.1177/002224299906300405

[B37] Ramaswamy V.. (2009). Co-creation of value—towards an expanded paradigm of value creation. Market. Rev. St. Gallen 26, 11–17. 10.1007/s11621-009-0085-7

[B38] Rosenbaum M. S.. (2009). Exploring commercial friendships from employees' perspectives. J. Serv. Market. 23, 57–66. 10.1108/08876040910933101

[B39] Rosenbaum M. S. Russell-Bennett R. Drennan J. (2015). Commercial friendships between gay sales associates and straight female customers in luxury settings: a proposed theoretical framework. J. Retail. Consum. Serv. 27, 179–186. 10.1016/j.jretconser.2015.08.004

[B40] Saha Gour C. Theingi. (2009). Service quality, satisfaction, and behavioural intentions: a study of low-cost airline carriers in Thailand. Manag. Serv. Qual. 19, 350–372. 10.1108/09604520910955348

[B41] Sato M. Jordan J. S. Funk D. C. (2014). The role of physically active leisure for enhancing quality of life. Leisure Sci. 36, 293–313. 10.1080/01490400.2014.88691217685922

[B42] Shain D. Chalasani S. (1992). Exploting niches using relation marketing. J. Mark. 3, 33–42. 10.1108/07363769210035215

[B43] Shen J. Zeng C. Lin Y. (2008). Study on the influence of tourists' tourists' leisure involvement and experience attachment – a case study of the Snake Kiln Pottery Cultural Park in Nantou, China. J. Human. Soc. Hsinchu Univ. Educ. 113–132.

[B44] Szymanski D. M. Henard D. H. (2001). Customer satisfaction: a meta-analysis of the empirical evidence. J. Acad. Market. Sci. 29, 16. 10.1177/0092070301291002

[B45] Tsai H.-T. Huang H.-C. (2007). Determinants of e-repurchase intentions: an integrative model of quadruple retention drivers. Inform. Manage. 44, 231–239. 10.1016/j.im.2006.11.006

[B46] Wang J. Wang D. Sun N. (2017). The influence of social clues on customer citizen behavior in network service scene – the role of continuous trust and business friendship. Soft Sci. 31, 112–116.

[B47] Wang T. Guo R. (2006). Research on the influence of commercial friendship on relationship quality and customer loyalty. Busin. Econ. Admin. 179, 35–41.

[B48] Westbrook R. A. Oliver R. L. (1981). Developing Better Measures of Consumer Satisfaction: Some Preliminary Results. ACR North American Advances.

[B49] Withall J. Jago R. Fox K. R. (2011). Why some do but most don't. barriers and enablers to engaging low-income groups in physical activity programmes: a mixed methods study. BMC Public Health 11, 1–13. 10.1186/1471-2458-11-507 21711514 PMC3141466

[B50] Wu M.. (2000). Statistical Analysis. SPSS Statistical Application Practice China Railway Publishing House.

